# Formulas calculating the reactance of tubular busbars and their derivation in primary electrical connection schemes

**DOI:** 10.1038/s41598-023-30408-2

**Published:** 2023-02-24

**Authors:** Qun Ge, Zaiqiang Li, Siyuan Liu, Jiaqi Xing

**Affiliations:** School of Electrical and Control Engineering, Liaoning Technical University, Huludao, 125105 China

**Keywords:** Engineering, Electrical and electronic engineering

## Abstract

Electrical switching operation in a substation which locates in a high-voltage transmission system alters operating modes of main wiring in either the substation or the system. Major alterations may have negative influences on the switchgear of main wiring in a short time. The quantitative study of this problem has to be based on establishing equivalent circuits of main wiring, when there rarely are formulas to calculate the reactance of tubular busbars. In this paper on the basis of the electromagnetic field theory, the magnetic induction and flux linkages outside and inside tubular conductors are obtained from the Ampere Loop Theorem, and then the formulas to calculate approximately the reactance of tubular busbars with a three-phase parallel arrangement are derived. From the process and results of the calculation in an example it may be seen that the formulas are applied simply, conveniently and rapidly, and may be valuably spread in practical electrical engineering.

## Introduction

The necessity of calculating reactance of busbars is discussed firstly.

High-voltage overhead or cable transmission lines are mostly constructed by flexible conductors, where their parameters and equivalent circuits have been adopted in power system analysis and calculation maturely^1–4^. Main wiring is an arrangement of busbar connection in power plants and substations. It is a key chain of a power system where busbars are mostly constructed by hard conductors (e.g., tubular busbars, etc.) and plays an important role in collecting and distributing electrical energy. Busbars are much shorter than transmission lines and connected to them in a perpendicular direction. When conducting an analysis and calculation in a power system, main wiring is modeled as voltage nodes, not considering the influence of the resistance and reactance of busbars on power distribution, etc.

Main wiring is one of the important factors affecting the reliability and flexibility of a power system. Changes of its operating modes and the maintenance of equipment in its switchgear are inevitably achieved by changing over switchgear’s states (e.g., a switch or disconnector is on or off), which is called switching operation. The process of switching operation alters the circuit connection formed by each electrical component in a power system and correspondingly operating parameters in it such as voltage, current, power, etc. When in power plants and substations are applied simple forms of main wiring (e.g., single bus wiring, etc.) or in complex forms of main wiring is carried out switching operation with few steps, the influence of above alteration of operating parameters on the normal steady-state operation of the power system may be ignored.

However, forms of main wiring of high reliability and flexibility with busbars (such as double bus wiring, etc.) are complex, and there is not only one practicable scheme for sequences and steps of switching operation. Taking changeover busbars in a form of double bus wiring for an example, even if the same kind of switching operation with the same initial operating mode is conducted, such as maintenance of busbars in operation, there are at least two schemes to realize make-before-break operation of busbar section disconnectors^5,6^: one is that making disconnectors connected to busbars in reserve in all bays before breaking disconnectors connected to busbars in operation in those bays; the other is make-before-break from one bay to another one by one, that is, making disconnectors connected to busbars in reserve in the first bay before breaking disconnectors connected to busbars in operation in the same bay, and then making and breaking in the second bay, in the third bay, etc. Different operation sequences form different circuit connections of main wiring, where it is possible to make current flowing through segments of busbars, switchgear, incoming and outgoing lines increase to be overcurrent temporally, which inevitably produces an influence on expected service lives of above-mentioned equipment. It will avoid the occurrence of short-term overcurrent phenomena in main wiring and build a theoretical foundation for the automated and intelligent development of switching operation to analyze and study the influence of its different sequences.

Taking main wiring as a research subject rather than the power system which includes the main wiring, a circuit model of busbars with resistance and reactance has to be established. The resistance calculation of busbar conductors has been discussed in detail in reference^1,7^, where the calculation method of their reactance parameter is missing.

Scholars, engineers and technicians in China have been researching and practising on the calculation of resistance and reactance of conductors. For example, in reference^8^ a simplified calculation formula of the reactance of rectangular busbars was derived by using the functional relation between the values of reactance of the conductors and their cross-sectional areas, and when the areas exceed 400 mm^2^ there occurred a large error. In reference^9^ the impedance values of conductors in bus ducts were calculated by using measured data. In reference^10^ a numerical calculation was carried out on the internal impedance of conductors with a rectangular cross-section, and a method to obtain their internal inductance was proposed by calculating magnetic induction using derivatives of a geometric mean distance and then calculating the magnetic energy using the Boundary Integral Method. In reference^11^ the impedance of long cylindrical conductors was rederived by using the Bessel function, and long conductors with various abnormal shapes were equivalent to the long cylindrical ones to calculate their impedance based on the electromagnetic theory. Considering a skin-effect, in reference^12^ closed-form formulas to calculate the internal impedance of solid and tubular cylindrical conductors were presented by using polynomial approximations of Bessel functions with large parameters.

A great of counterparts outside China have also performed much work in this area. Authors of references^13,14^ investigated the self-inductance of a long conductor and the inductance of a single-phase line with a rectangular cross-section respectively and proposed new exact closed formulae. In reference^15^ the same authors proposed a new numerical method of calculating the impedance of a rectangular busbar system. In reference^16^ an analytical method for calculating impedances of rectangular bus ducts was presented. An interesting vector synthesis method was proposed in reference^17^ to solve the stray inductance of the laminated busbars connected to capacitors with switching power modules. In reference^18^ a novel numerical approach based on the fast Fourier transform and the convolution theorem was proposed to model the rectangular conductors of the busbar system. In reference^19^ a novel numerical technique, i.e., the Proper Generalized Decomposition, for the calculation of dc and ac internal inductances of rectangular conductors was introduced.

In the above literature there is more to analyze and calculate impedance of rectangular conductors and conductors with irregular cross-sections in distribution networks with low-voltage levels than that of tubular busbars in transmission networks with high-voltage levels. Guided by the electromagnetic field theory, in this paper distribution of magnetic fields around tubular conductors is derived, a simplified formulation for calculating the reactance of tubular busbars in a three-phase parallel arrangement is obtained. This will supplement parameters and mathematical models of a power system with that of busbars accordingly and it is suitable to apply in some kinds of calculation and analysis in electrical engineering practices.

## Results

In this paper on the basis of the electromagnetic field theory, the magnetic fields around three-phase tubular busbars in a parallel arrangement have been analyzed, and the formulas to calculate their inductance and reactance have been derived. It is easy to understand the analysis of the magnetic fields and the derivation process of the formulas and convenient to apply.

## Methods

### Reactance of a single-phase tubular conductor

The reactance of a conductor will be calculated by its definition *x* = 2*πfL*. The self-inductance *L* is also derived by its definition. Assuming that current *i* flows in a circuit, after calculating magnetic induction *B* and flux linkage *ψ*, by the definition formula of self-inductance *L* there is^20–22^:

1 $$L = \frac{\psi }{i}$$

**Figure 1:**
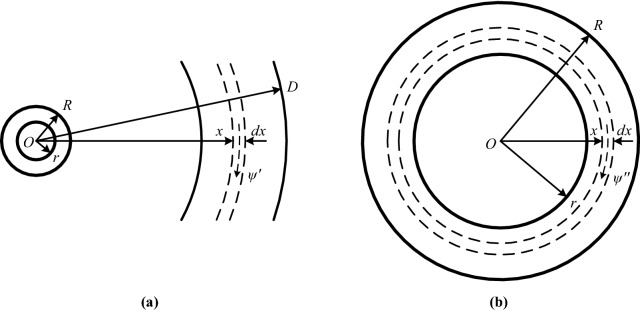
The distribution of magnetic fields of a tubular conductor: **(a)** external; **(b)** internal.

Two tubular conductors in a single-phase round-trip are constructed to be a single-turn coil, around which the distribution of magnetic fields is referred to the diagram of the single-phase wire in reference^1^. Letting one of the two conductors be at infinity, the magnetic flux around another is in the shape of concentric circles. This flux is constructed by the external one outside the conductor and the internal one inside it, and the distribution of magnetic fields is shown in Fig. 1a and b respectively. In Fig. 1 is also shown the cross-section of the conductor with center *O*, inner radius *r* and outer radius *R*.

#### Magnetic flux linkage outside the tubular conductor

In Fig. 1, letting current *i* flowing through the tubular conductor to be in uniform distribution, the flux linkage outside the conductor will be discussed firstly. Taking point *O* as the center, an integral loop with radius *x* (*x* > *R*) is made outside the conductor, as shown in Fig. 1a. Applying the Ampere Loop Theorem, there is

2 $$\oint\limits_{l} {{\varvec{B}}_{x}^{^{\prime}} } \cdot {\text{d}}{\varvec{l}} = B_{x}^{^{\prime}} 2\pi x = \mu_{x}{i}$$

where *B*_*x*_ʹ—the magnetic induction outside the tubular conductor; *μ*_*x*_—the magnetic permeability of the magnetic dielectric outside the tubular conductor, which is *μ*_*x*_ = *μ*_*r*_* μ*_0_; *μ*_*r*_—the relative magnetic permeability of magnetic dielectric, for the air there is *μ*_*r*_ = 1; *μ*_0_—the magnetic permeability of vacuums, there is

3 $$\mu_{0} = 4\pi \times 10^{ - 7} {\text{ H}}/{\text{m}}$$

Considering that the relative magnetic permeability *μ*_*r*_ of the air is 1, substituting *μ*_*r*_ = 1 and Eq. (3) into Eq. (2), the magnetic induction outside the conductor is obtained to be

4 $$B_{x}^{^{\prime}} = \frac{{\mu_{0} {i}}}{2\pi x} = 2 \times 10^{ - 7} \times \frac{{i}}{x} \left( {\text{T}} \right)$$

As shown in Fig. 1a, outside the conductor at point *x* is made a hollow cylinder with the thickness of *dx* and the length of 1 m, where the magnetic flux is equal to that passing through the area element *dS* = *dx* × 1. According to Eq. (4) and the definition of a magnetic flux, there is

5 $$d\Phi_{x}^{^{\prime}} = B_{x}^{^{\prime}} \cdot {\text{d}}S = 2 \times 10^{ - 7} \times \frac{{i}}{x} \times \left( {dx \times 1} \right) = 2 \times 10^{ - 7} \times i\frac{dx}{x}$$

The flux linkage corresponding to the magnetic flux in Eq. (5) surrounds the whole conductor, it is

6 $$d\psi_{x}^{^{\prime}} = d\Phi_{x}^{^{\prime}} \times 1 = 2 \times 10^{ - 7} \times i\frac{dx}{x}$$

Taking *D* and *R* as the upper and lower limits respectively, after Eq. (6) is integrated, the flux linkage (per unit length) at the radius of *D* outside the conductor surrounding the whole conductor is obtained to be

7 $$\psi^{\prime} = \int \limits_{R}^{D} {\text{d}}\psi_{x}^{^{\prime}} = \int \limits_{R}^{D} 2 \times 10^{ - 7} \times {i}\frac{{{\text{d}}x}}{x} = 2 \times 10^{ - 7} \times {i}\ln \frac{D}{R} \left( {{\text{Wb}}/{\text{m}}} \right)$$

#### Magnetic flux linkage inside the tubular conductor

As shown in Fig. 1b, taking point *O* as the center of circles, an integral loop with radius *x* (*r* ≤ *x* ≤ *R*) is made inside the tubular conductor. Applying the Ampere Loop Theorem, there is

8 $$\oint\limits_{l} {{\varvec{B}}_{x}^{^{\prime\prime}} } \cdot {\text{d}}{\varvec{l}} = B_{x}^{^{\prime\prime}} 2\pi x = \mu_{x} \frac{{\pi \left( {x^{2} - r^{2} } \right)}}{{\pi \left( {R^{2} - r^{2} } \right)}}{i}$$

where *B*_*x*_″—the magnetic induction inside the tubular conductor.

Considering relative magnetic permeability *μ*_*r*_ of the conductor and Eq. (3), the magnetic induction at the radius of *x* inside the conductor is obtained from Eq. (8) to be

9 $$B_{x}^{^{\prime\prime}} = \frac{{\mu_{r} \mu_{0} \left( {x^{2} - r^{2} } \right)}}{{2\pi \left( {R^{2} - r^{2} } \right)x}}{i} = 2 \times 10^{ - 7} \times \frac{{\mu_{r} i}}{{R^{2} - r^{2} }}\frac{{x^{2} - r^{2} }}{x} \left( {\text{T}} \right)$$

As shown in Fig. 1b, inside the conductor at point *x* is made a hollow cylinder with the thickness of *dx* and the length of 1 m, the magnetic flux inside the cylinder is equal to the magnetic flux passing through the area element *dS* = *dx* × 1. According to Eq. (9) and the definition of a magnetic flux, there is

$$d\Phi_{x}^{^{\prime\prime}} = B_{x}^{^{\prime\prime}} \cdot {\text{d}}S = 2 \times 10^{ - 7} \times \frac{{\mu_{r} i}}{{R^{2} - r^{2} }}\frac{{x^{2} - r^{2} }}{x}\left( {dx \times 1} \right)$$

10 $$= 2 \times 10^{ - 7} \times \frac{{\mu_{r} i}}{{R^{2} - r^{2} }}\frac{{\left( {x^{2} - r^{2} } \right)}}{x}dx$$

The flux linkage corresponding to the magnetic flux in Eq. (10) does not surround the whole conductor, but only a part of the conductor $\frac{{\pi \left( {x^{2} - r^{2} } \right)}}{{\pi \left( {R^{2} - r^{2} } \right)}}$, thus there is

11 $$d\psi_{x}^{^{\prime\prime}} = d\Phi_{x}^{^{\prime\prime}} \times \frac{{\pi \left( {x^{2} - r^{2} } \right)}}{{\pi \left( {R^{2} - r^{2} } \right)}} \times 1 = 2 \times 10^{ - 7} \times \frac{{\mu_{r} i}}{{\left( {R^{2} - r^{2} } \right)^{2} }}\frac{{\left( {x^{2} - r^{2} } \right)^{2} }}{x}dx$$

Taking *R* and *r* as the upper and lower limits respectively, after Eq. (11) is integrated, the flux linkage (per unit length) inside the conductor is obtained to be

12 $$\begin{aligned} \psi^{\prime\prime} & = \int \limits_{r}^{R} {\text{d}}\psi_{x}^{^{\prime\prime}} = \int \limits_{r}^{R} 2 \times 10^{ - 7} \times \frac{{\mu_{r} i}}{{\left( {R^{2} - r^{2} } \right)^{2} }}\frac{{\left( {x^{2} - r^{2} } \right)^{2} }}{x}dx \\ & = 2 \times 10^{ - 7} \times \mu_{r} i\left[ {\frac{{R^{2} - 3r^{2} }}{{4\left( {R^{2} - r^{2} } \right)}} + \frac{{r^{4} }}{{\left( {R^{2} - r^{2} } \right)^{2} }}ln\frac{R}{r}} \right] \left( {{\text{Wb}}/{\text{m}}} \right) \\ \end{aligned}$$

Letting the coefficient caused by the cross-sectional shape of the tubular conductor be

13 $$F_{tb} = \frac{{R^{2} - 3r^{2} }}{{4\left( {R^{2} - r^{2} } \right)}} + \frac{{r^{4} }}{{\left( {R^{2} - r^{2} } \right)^{2} }}ln\frac{R}{r}$$

Considering Eq. (13), the internal flux linkage of the conductor shown in Eq. (12) is simplified to be the following form:

14 $$\psi^{\prime\prime} = 2 \times 10^{ - 7} \times i\mu_{r} F_{tb} \left( {{\text{Wb}}/{\text{m}}} \right)$$

where *F*_*tb*_—the coefficient of cross-sectional shapes of tubular conductors calculated by Eq. (13).

At the distance of *D* from the center of the single tubular conductor the total flux linkage (per unit length) surrounding the whole conductor is the sum of the flux linkage in Eq. (7) and in Eq. (14), that is

15 $$\psi = \psi^{\prime} + \psi^{\prime\prime} = 2i\left( {ln\frac{D}{R} + \mu_{r} F_{tb} } \right) \times 10^{ - 7} \left( {{\text{Wb}}/{\text{m}}} \right)$$

#### Reactance of a single-phase tubular conductor

**Figure 2:**
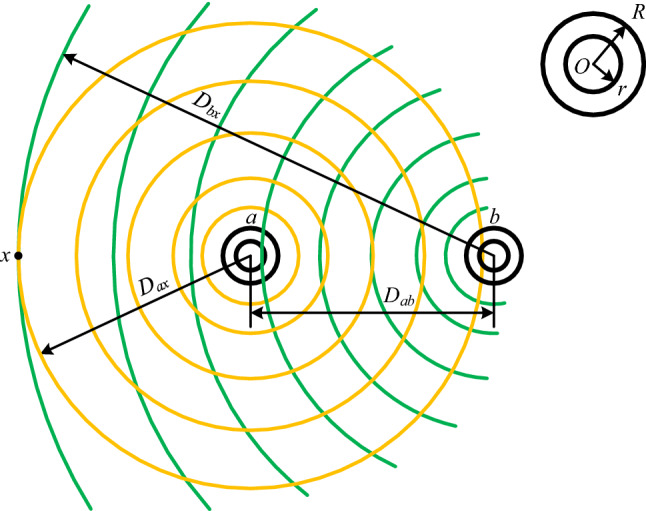
The distribution of magnetic fields of a single-phase conductor.

A single-phase coil composed of conductor *a* and conductor *b* is shown in Fig. 2. Taking conductor *a* as a reference, the distance of *b* from *a* is *D*_*ab*_, and *D*_*ab*_ >  > *R*. The yellow curves represent the flux lines generated by the current in conductor *a*, and the green ones – in conductor *b*.

In Eq. (15), after *D* is substituted by *D*_*ax*_ and *i* – by *i*_*a*_, the total flux linkage (per unit length) surrounding the conductor *a* generated by current *i*_*a*_ in conductor *a* at the distance *D*_*ax*_ from the center of the cross-section of conductor *a* is obtained to be

16 $$\psi_{a.ax} = 2i_{a} \left( {ln\frac{{D_{ax} }}{R} + \mu_{r} F_{tb} } \right) \times 10^{ - 7} \left( {{\text{Wb}}/{\text{m}}} \right)$$

In Eq. (7), after *D* is substituted by *D*_*bx*_, *R* – by *D*_*ab*_ and *i* – by *i*_*b*_, the flux linkage (per unit length) only winding around conductor *a* generated by current *i*_*b*_ in conductor *b* at the distance *D*_*bx*_ from the center of the cross-section of conductor *b* is obtained to be

17 $$\psi_{b.ax} = 2i_{b} ln\frac{{D_{bx} }}{{D_{ab} }} \times 10^{ - 7} \left( {{\text{Wb}}/{\text{m}}} \right)$$

According to the Superposition Principle, the total flux linkage winding around conductor *a* (per unit length) at straight line *x* parallel to the axis of conductor *a* (as shown in Fig. 2) is the sum of the flux linkage shown in Eqs. (16) and (17), that is

$$\psi_{ax} = \psi_{a.ax} + \psi_{b.ax} = 2\left[ {i_{a} \left( {ln\frac{{D_{ax} }}{R} + \mu_{r} F_{tb} } \right) + i_{b} ln\frac{{D_{bx} }}{{D_{ab} }}} \right] \times 10^{ - 7} \left( {{\text{Wb}}/{\text{m}}} \right)$$

Considering *i*_*b*_ =  − *i*_*a*_ in the single-phase conductor and substituting it into the above equation, there is

18 $$\psi_{ax} = 2i_{a} \left( {ln\frac{{D_{ax} D_{ab} }}{{RD_{bx} }} + \mu_{r} F_{tb} } \right) \times 10^{ - 7} \left( {{\text{Wb}}/{\text{m}}} \right)$$

When the straight line *x* is located at infinity, the above equation is exactly the total flux linkage (per unit length) surrounding the conductor *a*, where there is *D*_*ax*_ ≈ *D*_*bx*_. After it is substituted into Eq. (18), the total flux linkage (per unit length) winding around conductor *a* is obtained to be

19 $$\psi_{a} = 2i_{a} \left( {ln\frac{{D_{ab} }}{R} + \mu_{r} F_{tb} } \right) \times 10^{ - 7} \left( {{\text{Wb}}/{\text{m}}} \right)$$

Substituting Eq. (19) into Eq. (1), the inductance (per unit length) of single-phase tubular either of conductor *a* or conductor *b* is obtained to be

20 $$L_{a} = L_{b} = 2\left( {ln\frac{{D_{ab} }}{R} + \mu_{r} F_{tb} } \right) \times 10^{ - 7} \left( {{\text{H}}/{\text{m}}} \right)$$

Multiplying the inductance (per unit length) in Eq. (20) by the angular frequency of *ω* = 2*πf*, the (positive-sequence) reactance (per unit length) of single-phase tubular conductors is obtained to be

21 $$x_{1} = 4\pi f\left( {ln\frac{{D_{ab} }}{R} + \mu_{r} F_{tb} } \right) \times 10^{ - 7} \left( {{\Omega }/{\text{m}}} \right)$$

### Reactance of three-phase tubular busbars in a parallel arrangement

Equation (21) shows an approximate formula to calculate the reactance of single-phase tubular conductors. The reactance of three-phase tubular busbars in a parallel arrangement will be derived as follows.

**Figure 3:**
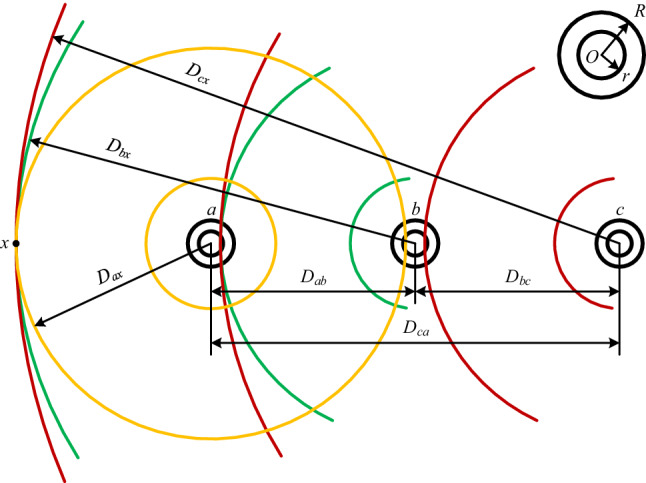
The distribution of magnetic fields of three-phase tubular conductors in a parallel arrangement.

The distribution of the magnetic fields of three-phase tubular conductors is shown in Fig. 3, where yellow, green and red curves represent the flux lines generated by the current in three-phase conductors *a*, *b* and *c* respectively. In Fig. 3*D*_*ab*_, *D*_*bc*_ and *D*_*ca*_ are the distances between the three-phase conductors respectively, and there are *D*_*ab*_ >  > *R*, *D*_*bc*_ >  > *R*, and *D*_*ca*_ >  > *R*.

Similar to the case of the single-phase conductor, the total flux linkage (per unit length) generated by current *i*_*a*_ in conductor *a* at the distance *D*_*ax*_ from the center of the cross-section of conductor *a* is expressed by Eq. (16), and the total flux linkage (per unit length) generated by current *i*_*b*_ in conductor *b* at the distance *D*_*bx*_ from the center of the cross-section of conductor *b* is expressed by Eq. (17).

Similarly, in Eq. (7), after *D* is substituted by *D*_*cx*_, *R* – by *D*_*ca*_ and *i* – by *i*_*c*_, the flux linkage (per unit length) only winding around conductor *a* generated by current *i*_*c*_ in conductor *c* at the distance *D*_*cx*_ from the center of the cross-section of conductor *c* is obtained to be

22 $$\psi_{c.ax} = 2i_{c} ln\frac{{D_{cx} }}{{D_{ca} }} \times 10^{ - 7} \left( {{\text{Wb}}/{\text{m}}} \right)$$

According to the Superposition Principle, the total flux linkage winding around conductor *a* (per unit length) at straight line *x* parallel to the axis of conductor *a* (as shown in Fig. 3) is the sum of the flus linkage in Eqs. (16), (17) and (22), that is

$$\begin{aligned} \psi_{ax} & = \psi_{a.ax} + \psi_{b.ax} + \psi_{c.ax} = 2\left[ {i_{a} \left( {ln\frac{{D_{ax} }}{R} + \mu_{r} F_{tb} } \right) + i_{b} ln\frac{{D_{bx} }}{{D_{ab} }} + i_{c} ln\frac{{D_{cx} }}{{D_{ca} }}} \right] \times 10^{ - 7} \\ & = 2\left[ {\left( {i_{a} lnD_{ax} + i_{b} lnD_{bx} + i_{c} lnD_{cx} } \right) + \left( {i_{a} ln\frac{1}{R} + i_{b} ln\frac{1}{{D_{ab} }} + i_{c} ln\frac{1}{{D_{ca} }} + i_{a} \mu_{r} F_{tb} } \right)} \right] \times 10^{ - 7} \\ \end{aligned}$$

When the straight line *x* is located at infinity from the three conductors *a*, *b* and *c*, there is *D*_*ax*_ ≈ *D*_*bx*_ ≈ *D*_*cx*_. Considering the normal steady-state operation of a power system, there is *i*_*a*_ + *i*_*b*_ + *i*_*c*_ = 0. Substituting these two conditions (*D*_*ax*_ ≈ *D*_*bx*_ ≈ *D*_*cx*_ and *i*_*a*_ + *i*_*b*_ + *i*_*c*_ = 0) into the above equation, after reorganizing it, the total flux linkage (per unit length) winding around conductor *a* is obtained to be

23 $$\psi_{a} = 2\left( {i_{a} ln\frac{1}{R} + i_{b} ln\frac{1}{{D_{ab} }} + i_{c} ln\frac{1}{{D_{ca} }} + i_{a} \mu_{r} F_{tb} } \right) \times 10^{ - 7} \left( {{\text{Wb}}/{\text{m}}} \right)$$

Similarly, the total flux linkage (per unit length) winding around conductor *b* and *c* are respectively obtained to be

24 $$\psi_{b} = 2\left( {i_{b} ln\frac{1}{R} + i_{c} ln\frac{1}{{D_{bc} }} + i_{a} ln\frac{1}{{D_{ab} }} + i_{b} \mu_{r} F_{tb} } \right) \times 10^{ - 7} \left( {{\text{Wb}}/{\text{m}}} \right)$$

25 $$\psi_{c} = 2\left( {i_{c} ln\frac{1}{R} + i_{a} ln\frac{1}{{D_{ca} }} + i_{b} ln\frac{1}{{D_{bc} }} + i_{c} \mu_{r} F_{tb} } \right) \times 10^{ - 7} \left( {{\text{Wb}}/{\text{m}}} \right)$$

The distances between the three-phase conductors in a parallel arrangement *D*_*ab*_, *D*_*bc*_ and *D*_*ca*_ are not completely equal. From Eqs. (23), (24) and (25) we know that three-phase flux linkages in normal operation are asymmetrical. For the sake of convenience in calculation, letting

26 $$D_{ab} \approx D_{bc} \approx D_{ca} \approx D_{eq} = \sqrt[3]{{D_{ab} D_{bc} D_{ca} }}$$

where *D*_*eq*_—the mutual geometric mean distance between the three-phase conductors.

Substituting Eq. (26) into Eqs. (23), (24) and (25) respectively, three-phase flux linkages (per unit length) in approximate symmetry are obtained to be

27 $$\left. {\begin{array}{*{20}c} {\psi _{a} = 2i_{a} \left( {ln\frac{{D_{{eq}} }}{R} + \mu _{r} F_{{tb}} } \right) \times 10^{{ - 7}} ~\left( {{\text{Wb}}/{\text{m}}} \right)} \\ {\psi _{b} = 2i_{b} \left( {ln\frac{{D_{{eq}} }}{R} + \mu _{r} F_{{tb}} } \right) \times 10^{{ - 7}} ~\left( {{\text{Wb}}/{\text{m}}} \right)} \\ {\psi _{c} = 2i_{c} \left( {ln\frac{{D_{{eq}} }}{R} + \mu _{r} F_{{tb}} } \right) \times 10^{{ - 7}} ~\left( {{\text{Wb}}/{\text{m}}} \right)} \\ \end{array} } \right\}$$

Substituting the three equations in Eq. (27) into Eq. (1) respectively, the inductance (per unit length) of three-phase conductors is obtained to be

28 $$L_{a} = L_{b} = L_{c} = 2\left( {ln\frac{{D_{eq} }}{R} + \mu_{r} F_{tb} } \right) \times 10^{ - 7} \left( {{\text{H}}/{\text{m}}} \right)$$

Multiplying the inductance (per unit length) in Eq. (28) by the angular frequency of *ω* = 2*πf*, the (positive-sequence) reactance (per unit length) of three-phase tubular conductors in a parallel arrangement is obtained to be

29 $$x_{1} = 4\pi f\left( {ln\frac{{D_{eq} }}{R} + \mu_{r} F_{tb} } \right) \times 10^{ - 7} \left( {{\Omega }/{\text{m}}} \right)$$

or

30 $$x_{1} = 4\pi f\left( {2.3lg\frac{{D_{eq} }}{R} + \mu_{r} F_{tb} } \right) \times 10^{ - 7} \left( {{\Omega }/{\text{m}}} \right)$$

The base of the logarithm in Eq. (29) is *e*, and in Eq. (30) it is 10.

## Examples

Two types of conductors used in power systems are selected, one is a three-phase overhead line of Aluminum Conductor Steel Reinforced (ACSR) conductors, another is a three-phase tubular bus of aluminum-magnesium alloy conductors. They are all arranged horizontally, and the distances between two phases are the same: 4 m, 4 m and 8 m. The outside diameter of each conductor of the overhead line is 30 mm (radius *R* is 15 mm). The model of the tubular busbars is *ϕ* 30 / 25 mm (outer radius *R* is 15 mm, inner radius *r* is 12.5 mm). The system frequency *f* is 50 Hz, the relative permeability of the conductors *μ*_*r*_ is 1.

The mutual geometric mean distance between the two three-phase conductors is the same, which is calculated by Eq. (26) and it is

$$D_{eq} = \sqrt[3]{4 \times 4 \times 8} = 5.039 68 \left( {\text{m}} \right) = 5 039.68 \left( {{\text{mm}}} \right)$$

The reactance (per unit length) of each phase of the three-phase overhead line is calculated by the following formula^1^:

31 $$x_{1} = 2\pi f\left( {2ln\frac{{D_{eq} }}{R} + \frac{{\mu_{r} }}{2}} \right) \times 10^{ - 7} \left( {{\Omega }/{\text{m}}} \right)$$

Substituting *f* = 50 Hz, *D*_*eq*_ = 5 039.68 mm, *R* = 15 mm, *μ*_*r*_ = 1 into Eq. (31), there is

$$x_{1} = 2\pi \times 50 \times \left( {2 \times ln\frac{5 039.68}{{15}} + \frac{1}{2}} \right) \times 10^{ - 7} = 3. 812 \times 10^{ - 4} \left( {{\Omega }/{\text{m}}} \right)$$

The value of coefficient *F*_*tb*_ caused by the cross-sectional shape of the conductors of the three-phase busbar is calculated by Eq. (13), substituting *r* = 12.5 mm, *R* = 15 mm into it, there is

$$F_{tb} = \frac{{15^{2} - 3 \times 12.5^{2} }}{{4 \times \left( {15^{2} - 12.5^{2} } \right)}} + \frac{{12.5^{4} }}{{\left( {15^{2} - 12.5^{2} } \right)^{2} }}ln\frac{15}{{12.5}} = 0.055 38$$

Substituting *f* = 50 Hz, *D*_*eq*_ = 5 039.68 mm, *R* = 15 mm, *μ*_*r*_ = 1, *F*_*tb*_ = 0.055 38 into Eq. (29), the reactance of the tubular busbar (per unit length) of each phase is obtained to be

$$x_{1} = 4\pi \times 50 \times \left( {ln\frac{5 039.68}{{15}} + 1 \times 0.055 38} \right) \times 10^{ - 7} = 3.689 8 \times 10^{ - 4} \left( {{\Omega }/{\text{m}}} \right)$$

From the results of the above two types of conductors with the same outside diameter and the same arrangement it may be seen, that the reactance (3.689 8 × 10^−4^ Ω/m) of the three-phase tubular busbars is a little smaller than the reactance (3.812 × 10^−4^ Ω/m) of the three-phase overhead lines.

## Data Availability

All data generated or analysed during this study are included in this published article.
